# An Analysis of Precipitation Isotope Distributions across Namibia Using Historical Data

**DOI:** 10.1371/journal.pone.0154598

**Published:** 2016-05-04

**Authors:** Kudzai Farai Kaseke, Lixin Wang, Heike Wanke, Veronika Turewicz, Paul Koeniger

**Affiliations:** 1 Department of Earth Sciences, Indiana University-Purdue University Indianapolis (IUPUI), Indianapolis, IN, 46202, United States of America; 2 University of Namibia, Geology Department, Private Bag 13301, Windhoek, Namibia; 3 University of Vienna, Department of Environmental Geosciences, Vienna, Austria; 4 Federal Institute for Geosciences and Natural Resources (BGR), Stilleweg 2, 30655, Hannover, Germany; Potsdam Institute for Climate Impact Research, GERMANY

## Abstract

Global precipitation isoscapes based on the Global Network for Isotopes in Precipitation (GNIP) network are an important toolset that aid our understanding of global hydrologic cycles. Although the GNIP database is instrumental in developing global isoscapes, data coverage in some regions of hydrological interest (e.g., drylands) is low or non-existent thus the accuracy and relevance of global isoscapes to these regions is debatable. Capitalizing on existing literature isotope data, we generated rainfall isoscapes for Namibia (dryland) using the cokriging method and compared it to a globally fitted isoscape (GFI) downscaled to country level. Results showed weak correlation between observed and predicted isotope values in the GFI model (r^2^ < 0.20) while the cokriging isoscape showed stronger correlation (r^2^ = 0.67). The general trend of the local cokriging isoscape is consistent with synoptic weather systems (i.e., influences from Atlantic Ocean maritime vapour, Indian Ocean maritime vapour, Zaire Air Boundary, the Intertropical Convergence Zone and Tropical Temperate Troughs) and topography affecting the region. However, because we used the unweighted approach in this method, due to data scarcity, the absolute values could be improved in future studies. A comparison of local meteoric water lines (LMWL) constructed from the cokriging and GFI suggested that the GFI model still reflects the global average even when downscaled. The cokriging LMWL was however more consistent with expectations for an arid environment. The results indicate that although not ideal, for data deficient regions such as many drylands, the unweighted cokriging approach using historical local data can be an alternative approach to modelling rainfall isoscapes that are more relevant to the local conditions compared to using downscaled global isoscapes.

## Introduction

Drylands are often defined on the basis of the ratio of mean annual precipitation to mean annual evaporative demand [1–3] and the aridity index (AI) is used to classify drylands as regions with AI < 0.65. Globally, they account for over 40% of the earth’s terrestrial surface [4] and are characterised by low and often seasonal rainfall resulting in permanent or seasonal soil water deficit [5] and ephemeral drainage. Despite limitations to dryland productivity due to water scarcity [6], they contribute approximately 40% of global net primary productivity [7] supporting more than 2 billion people worldwide [8, 9]. Global water resources are inherently related to and affected by population growth [10]. 90% of the dryland population resides in developing countries which have an above average population density growth, exacerbating the already tight limitations imposed by water availability and food security on these systems [3]. Therefore, there is a need to understand hydrological processes at both global and local scales to create an inventory of available water resources and encourage efficient management of these resources.

Stable isotopes (δ^18^O and δ^2^H) in precipitation and groundwater are valuable environmental tracers that can be used to understand dynamics and processes in hydrology, geology, ecology and climate research [11–13]. Isotope fractionation processes impart unique signatures on meteoric water that can be combined with deuterium excess (*d*), a second-order isotope parameter defined as *d* = δD– 8 x δ^18^O [14] to determine vapour source origins and evaporative conditions [15]. Points that fall on the Global Meteoric Water Line (GMWL) have a constant *d* of 10‰ because rainout isotopic fractionation is considered an equilibrium process. Since the effect of equilibrium Rayleigh condensation processes roughly follows the GMWL slope of 8, variations in *d* can provide information about the environmental conditions (e.g., relative humidity and temperature) during non-equilibrium processes in oceanic moisture source regions [16]. Stable isotope hydrology is therefore based on interpreting isotope variations (δ^2^H, δ^18^O and *d*) in precipitation which are governed by the origins and conditions during cloud formation and rainout [11], thus the distribution of precipitation isotopes measured from a particular location reflect the local temperature, latitude and altitude [14, 17, 18]. The relationship between water isotope ratios, meteorological and geographical parameters allows for the production of regional and global isotopic landscapes—isoscapes [19–22]. These isoscapes enable the documentation and visualization of large-scale hydrological processes and provide point estimates. The examination of deviant values from the trend surface can highlight values that are unusual in their geographic context [19]. However, recent research suggests that *d* can be significantly altered by local processes and is thus not a true reflection (conservative tracer) of humidity at the source region as previously assumed [16, 23, 24].

Understanding the spatio-temporal variation of precipitation patterns (isotopes) could provide further information on regional and global hydrologic processes that enable better planning and preparation for climate change [25]. The basis for most global isoscapes is the Global Network for Isotopes in Precipitation (GNIP) dataset, initiated by the International Atomic Energy Agency (IAEA) and the World Meteorological Organisation (WMO) in 1961. However, the dataset has a coarse spatio-temporal resolution in some areas and is also affected by station unevenness [26] resulting in insufficient data coverage for many regions that are of interest to hydrologists, geologists and ecologists [27, 28]. These inconsistencies in data continuity complicate efforts to evaluate seasonal and long-term trends in isotope distributions as accuracy and precision of geostatistical estimation is linked to data density. To overcome the temporal heterogeneity of data, studies have adopted the use of long-term average values rather than specific months or years thus greatly increasing the sampling density by integrating data across time [29]. The integration of data over time is achieved via two methods: weighted by amount approach [30] which gives a measure of the net flux of isotopes to the land surface [29] and the alternative unweighted approach [31] used as a last resort when handling historical data and amounts are scarce or not reported. However, regardless the method applied, data aggregation although advantageous in some circumstances will inevitably cause data reduction and details of data reduction should be carefully considered as they could potentially introduce random or systematic error within the reduced data set [29].

Modelling of global hydrologic isoscapes has evolved over the years [21, 30–32]; however, each of these models suffers some degree of uncertainty due to dataset problems mentioned previously. Additionally, isotope ratios show a strong correlation with mean annual temperature in nontropical regions [21], and global isoscapes do not always work well in the tropics explaining 58–61% of the global isotopic variance in precipitation [32]. Furthermore, there is often a mismatch between observed isotope data and modelled results when models are downscaled especially in data deficient regions (e.g., Africa, Asia and the topics) [32]. Researchers have employed various strategies to reduce the mismatch between the observed data and model results at regional or country level. These approaches include constraining the geographical extent of the models and adding meteorological data as explanatory variables to improve interpolations at local scales [32–36].

The Regionalised Cluster-based Water Isotope Prediction (RCWIP) model [32, 36] is a recent precipitation isoscape model which out performs traditional modelling approaches 67% of the time. However, the RCWIP model [32, 36] coverage is limited to specific regions thus for many regions we still have to rely on the fixed regression-interpolation models of global rainfall isoscapes such as the Bowen and Wilkinson model [19, 30]. Spatial interpolation allows estimation of the isotopic composition of precipitation where data is not available by generating a smoothed trend surface that captures the geographic variability of the data. Therefore, despite the inherent problems of the GNIP dataset, improvements in model processing and data interpolation techniques make it a valuable resource and starting point to understanding hydrological isoscapes and processes at a global scale. However, refinement of these models at a local scale is necessary especially in data scarce regions as these models may fail to capture important local trends due to the coarse resolution of the models.

The goal of this study is to develop precipitation isoscapes that reflect local meteorological and physical conditions of a typical arid environment (Namibia) based on historical data from different isotope studies and use the generated isoscapes to address the following questions: 1) what are the differences between the globally fitted isoscape (GFI) and our precipitation isoscape? 2) is there a coherent spatial pattern in precipitation isoscapes across Namibia and what are the mechanisms responsible for the spatial pattern?

## Materials and Methods

### Study Region

**Fig 1:**
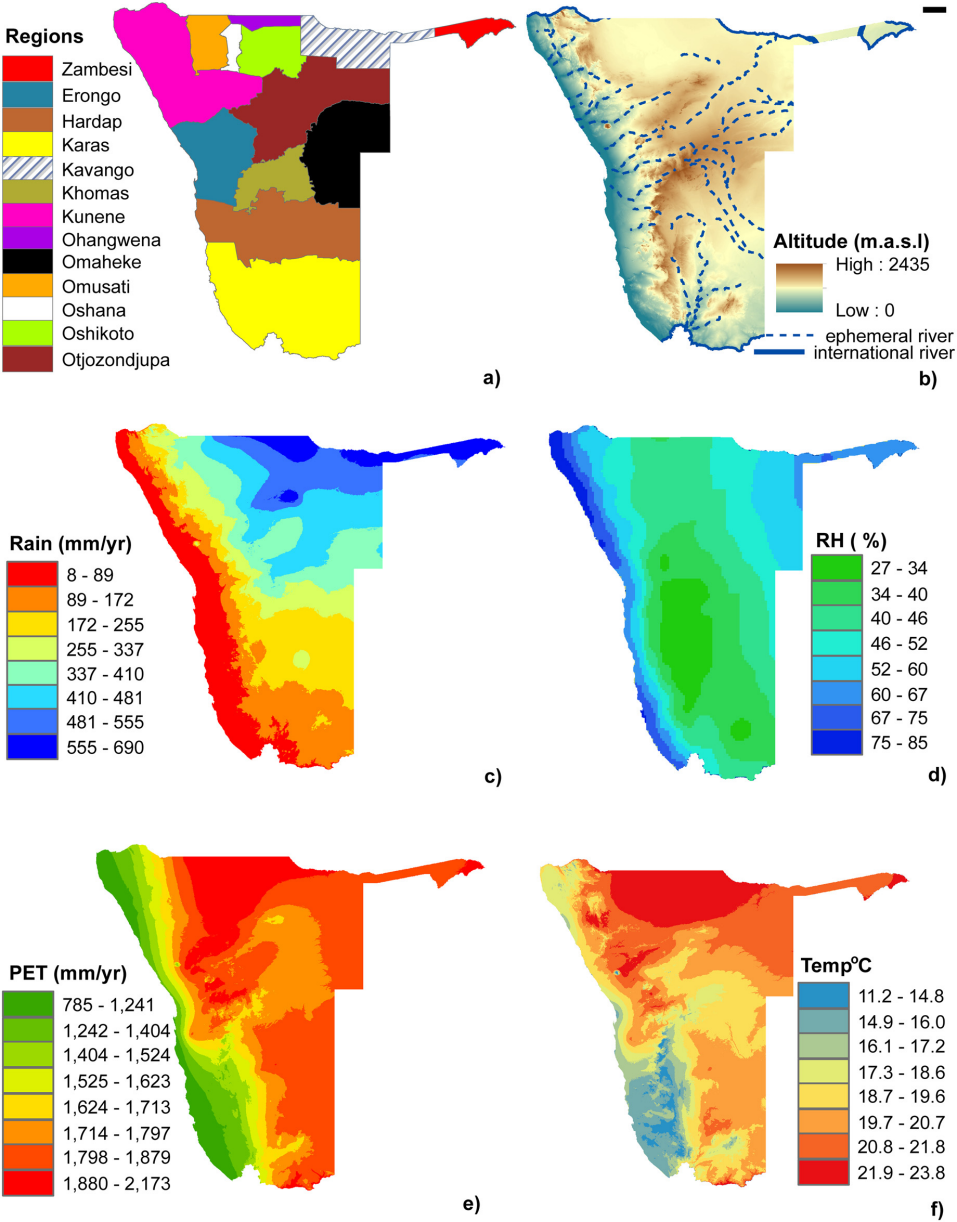
Geographical and meteorological data for Namibia. (a) Namibia’s administrative regions, (b) digital elevation model (DEM), (c) mean annual rainfall, (d) mean annual relative humidity (RH%), (e) mean annual potential evapotranspiration (PET) and (f) mean annual temperature.

Namibia is located on the south-western tip of the Southern African subcontinent. The country covers an area of over 825 000 km^2^ but is represented by only two stations in the GNIP network. Its position is strongly influenced by the aridifying nature of the cold Benguela current and dry descending air of the Global Hadley Circulation which limits convectional rainfall throughout much of the country’s interior [37, 38]. Namibia is officially classified as dryland [39], although climate varies from arid and semi-arid in the west to a more subtropical summer-rainfall climate in the north-east [37] (Fig 1c). The hyper arid Namib Desert stretches over 2000 km, from southern Angola through Namibia into South Africa with a variable width (80–200 km) and gradual rise from the Atlantic coast to the foot of the Namib Escarpment [40]. The Namib Escarpment runs north to south along the Atlantic coast and is characterised by a steep elevation gradient to over 2000 m (Fig 1b). Fig 1 shows the administrative geographical regions and some physical and meteorological parameters across Namibia.

### δ^18^O and δ^2^H data sources and data processing

We compiled a database of rainfall isotopic studies conducted in Namibia from a literature review from 1960–2010 [41]. Additional isotopic data for the Central Namib Desert at Gobabeb Research Centre was collected from a 2014 field campaign, where samples were collected immediately after the rain event, stored in vials and analysed using laser spectrometery (SD 0.2 ‰ δ^18^O and 1.1‰ δ^2^H) [42]. We acknowledge discontinuity of the data and that the studies often differed in their scope and analysis methods but the fundamental principles remained constant. Data was and is reported in δ notation relative to VSMOW-SLAP scale defined as,

$$\delta ‰=\left(\frac{R_{sample}}{R_{standard}}-1\right)x\;10^{3}$$

where R_sample_ and R_standard_ are the molar ratios of heavy to light isotopes (^2^H/H or ^18^O/^16^O) of the sample and standard.

Given the limited availability of isotopic data in this region, and that most of this historical data did not report rainfall amounts, we made an effort to integrate and conserve as much data as possible by adopting the unweighted approach [31]. Before adopting the unweighted approach, we discarded data when either δ^18^O or δ^2^H was not reported for a site or when geographic coordinates were not provided. Because the southern and south-eastern parts of Namibia were inadequately represented in the database, we incorporated data from nearby studies conducted in Botswana and South Africa to increase robustness of the database (Cape Town, Wolkop, Uitkyyk, Twatuin [43] and Lobastse [44]). The final rainfall database consisted of 45 locations (40 in Namibia and 5 outside, S1 Table).

### Meteorological data

A matrix of physical and meteorological variables plausibly related to precipitation isotope ratios were obtained at 30 arc second raster resolution for these variables. These variables included: elevation, mean annual precipitation, mean annual temperature, minimum temperature, maximum temperature [45], mean annual potential evapotranspiration (PET), Aridity Index (AI) [46] and mean annual relative humidity (RH) [47]. Data from each of these raster datasets was extracted for each of the 45 data points and we also calculated the straight-line distance from each data point to the Atlantic Ocean (S1 Table).

### Isoscape cokriging

Models were generated based on multiple regressions of location-based rainfall isotopes and associated physical and meteorological data followed by cokriging (interpolation) of the best performing models [26, 32]. The meteorological and physical data extracted from the raster datasets were used as predictors for the isoscapes and we did not compute non-linear combinations with the exception of the multiplicative combination of elevation and distance to the coast as we expected significant interaction between the two [26]. We performed exploratory regression of the data followed by ordinary least squares (OLS) regression which assessed the model residuals for normalcy, the major assumption of the statistical methods employed. The passing models were recalculated using geographic weighted regression analysis (GWR) to improve model performance and ranked using the Akaike Information Criterion (AICc). The AICc enables model selection by balancing r^2^ and simplicity thus giving an estimate of the quality of the model.

**Fig 2:**
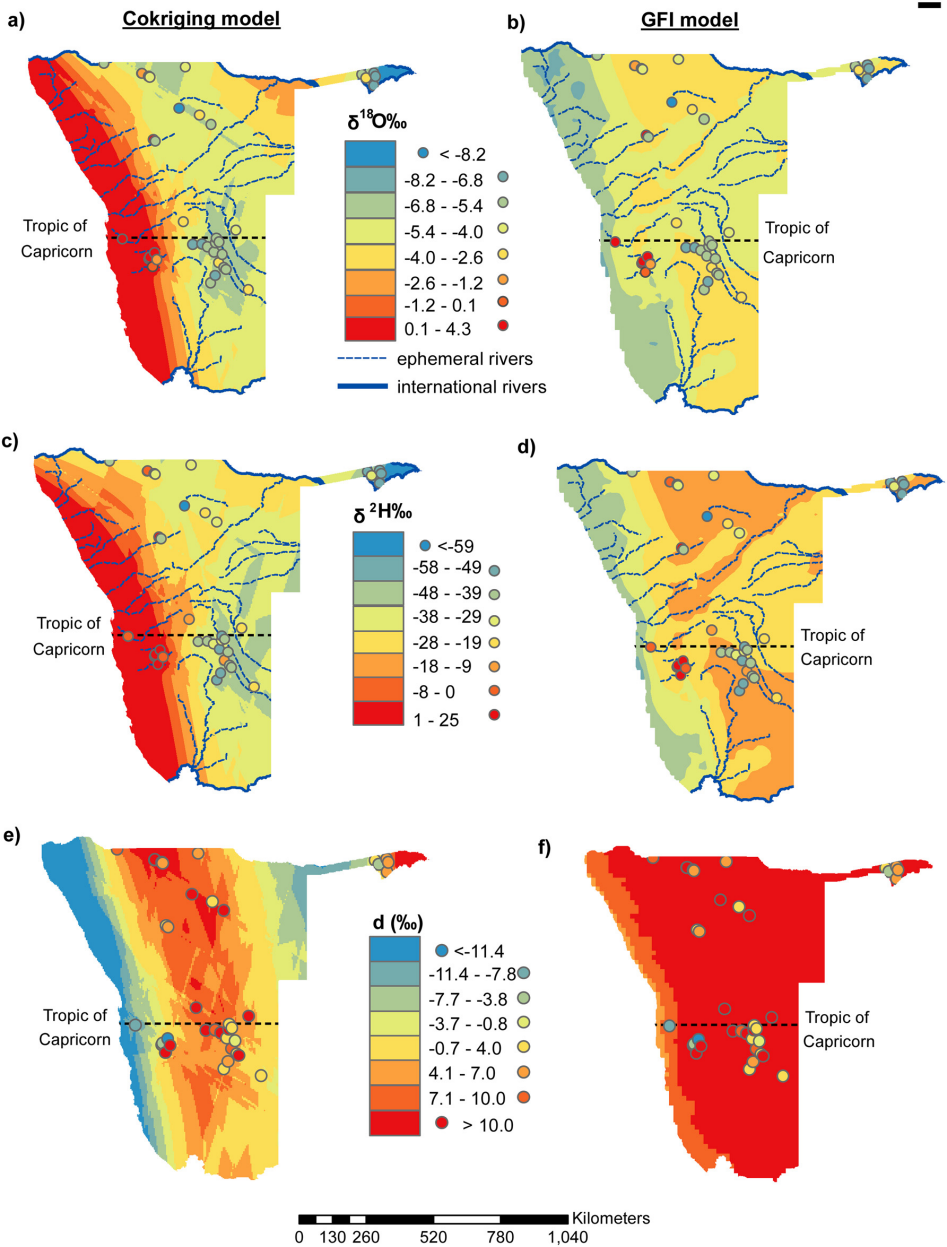
Rainfall cokriging isoscapes and the globally fitted isoscape (GFI) overlain with observed data. (a) δ^18^O cokriging isoscape, (b) δ^18^O GFI, (c) δ^2^H cokriging isoscape, (d) δ^2^H GFI, (e) d-excess (*d*) cokriging isoscape, and (f) d-excess (*d*) GFI.

We selected the top five models (Table 1) and looked for the highest ranking models that appeared in both δ^18^O and δ^2^H, based on the AICc (highlighted in bold Table 1). Selection of the highest ranking models common to both δ^18^O and δ^2^H would ensure no strange artifacts when cokriging was done to produce the *d* isoscape [26] as *d* integrates δ^18^O and δ^2^H. The highlighted models (Table 1) were then used in the ordinary cokriging interpolation to produce rainfall δ^18^O and δ^2^H isoscapes using ArcGIS 10.2.2 (Fig 2a and 2c). The Gaussian kernel function (goodness of fit = 4.17) was used and anisotropy accounted for in the interpolation process, mean error (0.98), mean standardised error (0.37), root-mean-square-error (6.08), average-standard error (2.57). Because the root-mean-square-error is greater than the average-standard error, variability was likely under-estimated because spatial distribution of observations was uneven as determined by the availability of data. The *d* isoscapes (Fig 2e and 2f) were calculated using the formula *d* = δD– 8 x δ^18^O [14] from the δ^18^O and δ^2^H isoscapes and were similar to those obtained from cokriging the best performing model (Table 1). We then restricted the geographic extent of the precipitation cokriging isoscapes to Namibia after interpolation. We downloaded the RCWIP model [32, 36] and restricted it to the geographic extent of Namibia and according to the model, this portion actually represents a globally fitted isoscape (GFI). We thus made a side by side comparison of our precipitation cokriging isoscape to the GFI for the geographic extent of Namibia overlain with the observed precipitation isotope data (Fig 2).

**Table 1:** Model parameters and goodness of fit statistics for regression models of predictive environmental variables. The model selected for interpolation and generation of a predictive surface (cokriging) for rainfall isoscapes are indicated in bold.

Isoscape	Parameters			OLS Model			GWR Model		
				AICc	r^2^	Rank	AICc	r^2^	Rank
**δ**^**18**^**O rain**	**elev. x coast**	**RH**		**215.5**	**0.34**	**1**	**214.7**	**0.36**	**1**
δ^18^O rain	elev. x coast	PPT		222.1	0.24	4	216.2	0.38	2
δ^18^O rain	PET	PPT		217.8	0.30	2	217.8	0.31	3
δ^18^O rain	PET	RH		224.0	0.20	5	219.6	0.28	4
δ^18^O rain	MT	RH	elev.	221.5	0.27	3	221.5	0.27	5
**δ**^**2**^**H rain**	**elev. x coast**	**RH**		**397.8**	**0.33**	**3**	**392.6**	**0.43**	**2**
δ^2^H rain	elev. x coast	PPT—PET	RH	392.5	0.42	1	392.4	0.42	1
δ^2^H rain	PET	RH		394.1	0.38	2	394.1	0.38	3
δ^2^H rain	PET	coast		395.9	0.35	4	395.4	0.36	4
δ^2^H rain	MT	coast		398.0	0.32	5	1221.0	0.63	5

Note: PPT-PET—precipitation minus potential evapotranspiration; MAP—mean annual precipitation; RH—relative humidity, Elev. x coast—elevation x distance to coast; T—mean annual temperature; Elev.—elevation; PPT—mean annual precipitation; OLS—ordinary least regression; GWR—geographic weighted regression.

## Results and Discussion

### Cokriging models for rainfall isotopes across Namibia

Precipitation across terrestrial Southern Africa originates as vapour from the Indian and Atlantic Oceans [48] and weather synoptics in this region are heavily influenced by the unique geomorphology and landmass orientation relative to the southern hemisphere’s atmospheric system [49]. The influence of the Namibian geomorphology and landmass orientation on rainfall is evident by the dominance of elevation (Namib Escarpment) and distance from the Atlantic coast on the rainfall models. The two best-performing models out of the top five δ^2^H and δ^18^O precipitation models have the elevation and distance to the coast parameters (Table 1 and Fig 1c) which suggests orographic rainfall patterns. The best performing model common to both δ^2^H and δ^18^O was selected to enable calculations of *d*, which would be affected if different models were used to calculate δ^2^H and δ^18^O isoscapes resulting in strange artifacts. The selected models used for cokriging are highlighted in Table 1 and shown in Fig 2.

### Comparison and interpretation of the cokriging and GFI isoscapes

#### Atlantic Ocean maritime vapour (Westerly winds)

The cokriging model predicts rainfall isotopic depletion in both δ^18^O and δ^2^H from several directions (Fig 2) suggesting that rainfall across the Namibian landscape is influenced by several synoptic weather systems. The cokriging model predicts δ^18^O and δ^2^H enrichment along the west coast with progressive depletion inland (Fig 2a and 2c). Rainfall isotopic gradients show a negative correlation with increasing distance from the vapour source due to progressive rainout also known as the ‘continental effect’ [14, 50, 51]. Therefore, the cokriging model suggests that some rainfall over terrestrial Namibia originates from the Atlantic Ocean and is progressively isotopically depleted as it moves inland (Fig 2a and 2c).

Although the ‘continental effect’ could be used to explain the rainfall isotopic trend predicted by the cokriging model from the coast inland, the degree of isotopic enrichment predicted and observed along the west coast is extreme and could be indicative of secondary processes affecting the isotopic signature (Fig 2a and 2c). Rain drops can re-evaporate below the cloud if in disequilibrium with the surrounding atmospheric humidity and the resulting kinetic fractionation effects can lead to enrichment of δ^18^O and δ^2^H in the droplets [52, 53]. Sub-cloud evaporation is common in short lived rain events where sub-cloud humidity does not achieve equilibrium with the falling droplets before the storm is over [53, 54]. According to the cokriging isoscape the most extreme enrichment in δ^18^O and δ^2^H rainfall isotopes is confined to the hyper-arid Namib Desert (Fig 2a and 2c) where annual precipitation is variable, 50–100 mm in the south, 5–18 mm in the Central Namib and < 50 mm in the north [38]. Low annual rainfalls suggest short-lived events which would create conditions that would be favourable for further isotopic enrichment due to sub-cloud evaporation hence the extreme degree of enrichment predicted and observed in the cokriging model and the observed isotopic values.

Rainfall in the Namib Desert is spatially and temporally variable although a general decreasing rainfall gradient east to west has been reported [55–58]. The isotopic range predicted by the cokriging model for the Namib Desert (0.1–4.3 ‰ δ^18^O and 1–25‰ δ^2^H) probably reflects this gradient and the ‘continental effect’ (Fig 2a and 2c). However, beyond the eastern edge of the Namib there is a narrow region of rapid isotopic depletion which coincides with the Namib Escarpment (Figs 1b, 2a and 2c). The steep elevation changes from the Namib Desert to the Namib Escarpment cause orographic lift of the Atlantic maritime air mass resulting in orographic rainfall over the region. The west to east gradient extends beyond the Namib Desert to the interior Central Plateau [38] and this is captured by the cokriging model which shows continued δ^18^O and δ^2^H depletion over the Namib Escarpment (Fig 2a and 2c).

#### Indian Ocean maritime vapour (Easterly winds)

The GFI model generally predicts a rainfall isotopic depletion gradient from east-west, with the most depleted values occurring along the Atlantic Coast in contrast to the cokriging model (Fig 2b and 2d). Because the GFI predicts isotopic depletion from east-west, this suggests Indian Oceanic origins of the maritime vapour which is progressively depleted and modified along its trajectory towards the Namibian coast due to rainout and evapotranspiration (Fig 2b and 2d). Summer rainfall over Southern Africa is dominated by cold dry westerlies flowing over warm moist easterlies which create instability and thunderstorms over South Africa, central and southern Botswana and central and south-east Namibia [59]. Furthermore, it has been indicated that at latitudes greater than 18°S in Namibia, most rainfall is supplied by the slow westward advance of tropical easterly disturbances peaking in February and March [60]. However, these storms seldom reach the west coast bordered by the eastern edge of the Namib Escarpment and are thus confined to the Central Plateau [38] in contrast to the depictions of the GFI model predictions (Fig 2b and 2d). This is because by the time the easterly winds get to the Namib Escarpment they would have lost most of their moisture as they advect over terrestrial Southern Africa resulting in warm, dry winds incapable of cloud formation and generating rain. Therefore the GFI model could be exaggerating the extent of the influence of the easterly winds on rainfall dynamics over Namibia (Fig 2).

The cokriging model on the other hand, predicts a similar east-west depletion in both δ^18^O and δ^2^H precipitation signatures but differs from the GFI model in that the predicted isotopic depletion does not extend all the way to the coast (Fig 2). According to the cokriging model, the Indian Ocean maritime vapour generated rainfall (convectional) exhausts before the Namib resulting in the most isotopically depleted rainfalls from this system occurring in the Khomas and central Hardap regions due to rainout effects (Fig 2a and 2c). The resulting warm dry winds develop into berg winds as they continue on a westward trajectory into the Namib Desert where they supress the convectional rise of the underlaying cool humid Atlantic vapour advecting inland, preventing cloud formation and rain [61]. This partially explains the low rainfall observed over the Namib Desert despite its close proximity to its rainfall vapour source, the Atlantic Ocean (Fig 1c). The cokriging isoscapes also predict convergence of the westerly (orographic) and easterly (convectional) derived rainfall over the same areas in the Central Plateau resulting in the most isotopically depleted rainfall occurring in the Khomas and central Hardap regions (Fig 2a and 2c). Therefore, according to the cokriging model convectional rainfall will not extend beyond these locations and vice versa for the orographic rainfall, which is in agreement with an earlier study [38]. Furthermore, observed rainfall isotopic values are similar to those predicted in the cokriging than the GFI model suggesting that the latter model could be overestimating the extent of the influence of the Indian Ocean on rainfall patterns in the Namib Desert (Fig 2a and 2c).

#### Zaire Air Boundary (ZAB) and the Intertropical Convergence Zone (ITCZ)

Rainfall in the wetter northern parts of Namibia is associated with the southward migration of the Intertropical Convergence Zone (ITCZ) and the Zaire Air Boundary (ZAB) which extends to about 18°S between January and February [62]. This is defined as a convergence of tropical and subtropical circulation over central and southern Africa associated with convective activity, cloud formation and precipitation [63, 64]. The ZAB system generates rainfall that extends into the northern parts of Namibia from Angola hence the high rainfall observed (Fig 1c) and the depleted signatures in the Oshana, Oshikoto and Ohangwena regions which can be attributed to rainout effects [14] (Fig 2a and 2c). Convergence of the ZAB and ITCZ during the austral summer generate rains that could be responsible for the north-east to south-west rainfall gradient across Namibia (Fig 1c). These convective thunderstorms enter Namibia via the Kavango region hence the relatively enriched isotopic signature but this is not as enriched as that observed over the Namib Desert because kinetic fraction effects are minimised due to the higher rainfall (Fig 1c). The rains decrease in a south-west direction which is reflected by the progressive radial depletion of the isotopic signatures predicted in the cokriging model (Fig 2a and 2c). The most depleted signatures are observed in the Zambesi region and this can be attributed to the amount effect (Fig 2a and 2c) as this region receives the highest rainfall in Namibia (Fig 1c).

#### Tropical Temperate Troughs (TTTs)

The TTTs are considered as the dominant summer rainfall producing system over southern Africa [65–67] accounting for about 39% of mean annual rainfall over the region [68]. Tropical Temperate Troughs link an easterly wave in the tropics to a westerly wave in the in the south [63] an event associated with a cloud band and precipitation [67]. Development of a continental low over central southern Africa in late summer enhances low level westerly flow from western southern Africa at 10°S facilitating anomalous water vapour convergence over eastern southern Africa [67]. This links with the TTT cloud band, such that the resulting rain extends further west over the region including Namibia than early summer [67]. The cokriging isoscapes depict an area of isotopically depleted rainfall in a general northwest-southeast direction which could be reflecting the cloud band and resulting rainfall from the TTTs (Fig 2a and 2c). The GFI model also appears to depict the influence of the TTTs over Namibia but in this case we observe isotopic enrichment in the northwest and southeast regions which does not match the observed data (Fig 2b and 2d).

### Modelled isotopic relationships

**Fig 3:**
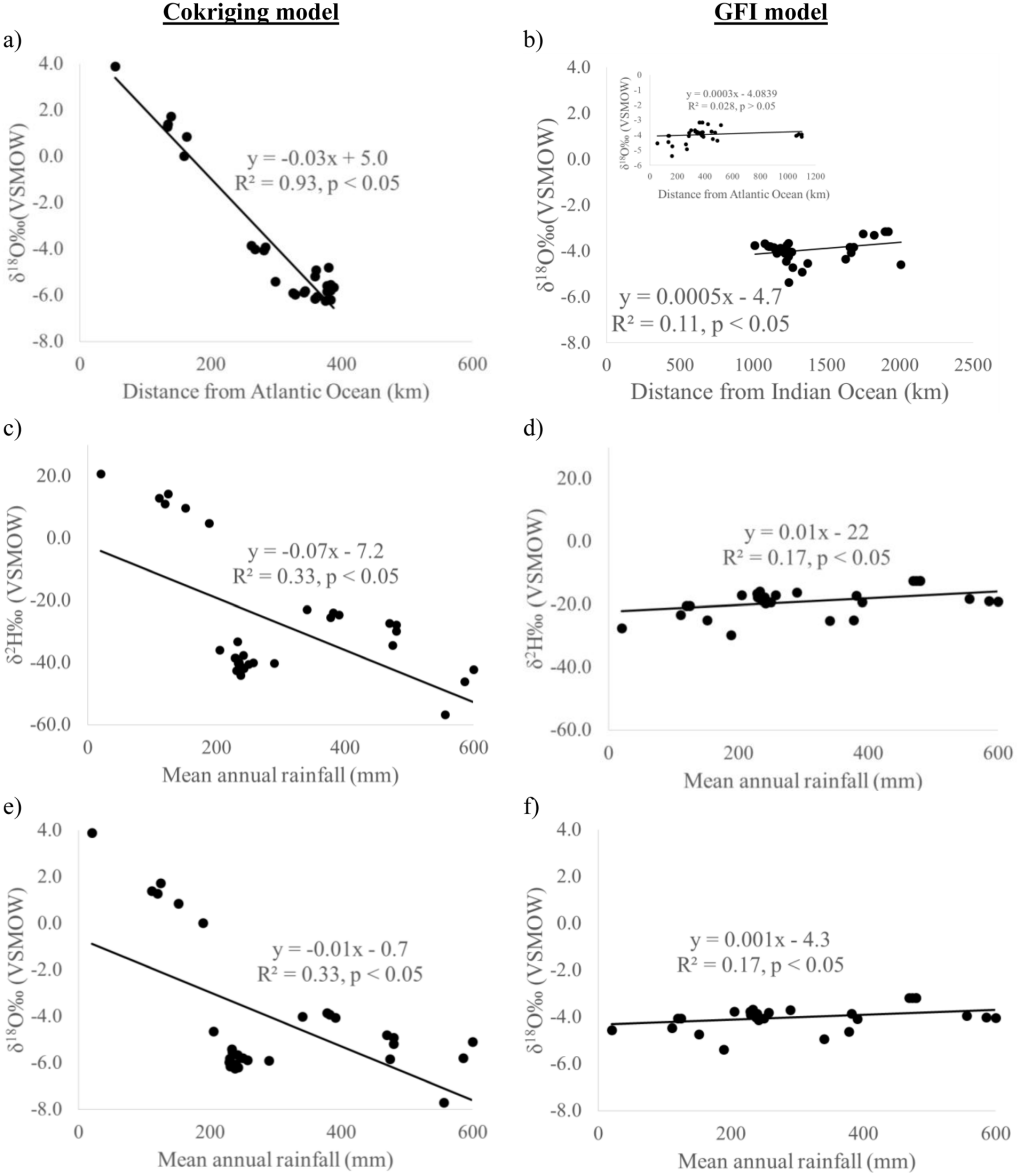
Modelled rainfall isotope relationships with distance from coast (continental effect) and rainfall amount (amount effect) across Namibia. (a) δ^18^O cokriging model ‘continental effect’, (b) δ^18^O GFI model ‘continental effect’, (c) δ^2^H cokriging model ‘amount effect’, (d) δ^2^H GFI model ‘amount effect’, (e) δ^18^O cokriging model ‘amount effect’, and (f) δ^18^O GFI model ‘amount effect’.

By extracting modelled isotopic values from both isoscapes we can investigate the classic isotope relations such as the ‘continental’ and ‘amount’ effects across the Namibian landscape (Fig 3). The “amount effect” is the negative relationship between precipitation isotopes and the amount of precipitation [14] and is observable at intra-seasonal or longer timescales [69]. Although the “amount effect’ is predominately due to sub-cloud evaporation and the recycling of the sub-cloud vapour layer [69] combining the two gives the opportunity to evaluate our modelled relationships. Given that the cokriging isoscape suggests that some orographic rainfall across Namibia originates in the Atlantic in a general west-east direction (Fig 2a and 2c), we modelled the ‘continental effect’ by calculating distance from the Atlantic Ocean to the extent of the Atlantic influence on rainfall isotopes as determined by the cokriging isoscape (Fig 2a and 2c). Fig 3a shows a strong negative correlation (r^2^ = 0.93, p < 0.05) between isotopic value and distance travelled inland from the Atlantic Ocean until the Central Plateau. This can be attributed to progressive rainout or ‘continental effect’ [14, 50, 51], thus Fig 3a supports the Atlantic origins of orographic rainfall over the western sections of Namibia as depicted in the cokriging isoscapes (Fig 2a and 2c). We did not consider the ‘continental effect’ from the Indian Ocean for the cokriging isoscape because the east-west rainfall isotope gradient extends to about 400 km in Namibia while distance to the Indian Ocean is over 1000 km. Therefore, the rainfall isotopic signature would certainly have been altered before reaching Namibia because of the distance involved [16, 23].

Given that the GFI model predicts a general east-west rainfall isotopic depletion gradient (Fig 2b and 2d), we did not expect to observe the “continental effect” from the Atlantic coast inland. The inset in Fig 3b confirms that there was no significant correlation between rainfall isotopic signature and distance from the Atlantic coast (p > 0.05). We thus evaluated the ‘continental effect’ using the distance from the Indian Ocean for the GFI model (Fig 3b). Fig 3b does not show the ‘continental effect’ instead showing a weak positive correlation (r^2^ = 0.11, p < 0.05) between isotopic signatures and distance from the Indian Ocean. The ‘continental effect’ is not always pronounced even in regions with strong rainfall gradients en route as reported for the Amazon [70]. This could be due to the return flux of moisture by transpiration and this invalidates the effect of rainout in subsequent rains further inland [71]. However, in the case of the GFI model the ‘continental effect’ is masked or interrupted by the enrichment observed in the north and south central portions of Namibia probably related to the TTTs (Fig 2b and 2d). Therefore, although we can observe a general east-west rainfall isotopic gradient on the GFI model, it is difficult to model the ‘continental effect’ from the Indian Ocean because of the influence of the TTTs and the distance involved which result in possible alterations of the isotopic signature.

Extracting the modelled isotopic values from both the cokriging and GFI models (Fig 2) we evaluated the ‘amount effect’ (Fig 3c–3f). The cokriging model shows a negative correlation between rainfall isotopic signature and rainfall amount (Fig 3c and 3e), the classic ‘amount effect’. The GFI model on the other hand depicts the opposite trend, a positive correlation between isotopes and annual rainfall contrary to expectation (Fig 3d and 3f). This suggests that the GFI model could be flawed in this region or could be reflecting convective precipitation which cannot be adequately explained by the ‘amount effect’ [72]. In addition, the opposite slope in GFI models could be related to the insufficient representation of topography at local scale. For example, higher latitudes tend to have less precipitation and lower delta values and vice versa on an annual basis, an effect opposite to the amount effect. Therefore if the topography was not represented in sufficient detail, this latitude effect may mask the “amount effect”.

### D-excess, *d*

The *d* cokriging isoscape depicts extremely low values along the west coast of Namibia (Fig 2e) and this could be related to the negative correlation between *d* and RH [73–75] (Fig 1d). The high RH over the Namib Desert can be attributed to the frequent fog occurrences along Namibia’s west coast [76] that penetrate to about 60 km inland [77] and their dissipation downwind could result in the observed high RH beyond the fog zone. Meanwhile, the rainfall gradient decreases from east to west [55–58] (Fig 1c) contrary to the RH trend. Therefore, the low *d* over the Namib Desert could be due to the negative correlation between *d* and RH from the frequent fog and its dissipation in this area (Fig 2e). At the same time, because δ^18^O and δ^2^H enrichment along the coast was attributed to sub-cloud evaporation (Fig 2a and 2c) this also means the low *d* could be also attributed to sub-cloud evaporation of falling raindrops through an unsaturated air column [14, 50, 52] as *d* = δD– 8 x δ^18^O [14]. Therefore, *d* in this area could be a result of the combined effects of RH (fog induced) and sub-cloud evaporation, depending on the prevailing conditions during the particular rainfall event.

The cokriging *d* isoscape also depicts extremely low values in the north eastern regions of Namibia (Kavango, Otjozondjupa and Omaheke) (Fig 2e). Because these regions have a subtropical climate [37] and high rainfall (Fig 1c), the prospects of sub-cloud evaporation are minimal. Therefore the low *d* values are unlikely caused by sub-cloud evaporation and they could be related to the negative correlation between *d* and RH [73–75]. The high RH in this region is due to evapotranspiration from the abundance of vegetation, which are supported by the higher rainfall in this area (Fig 1c and 1d). We, however, acknowledge the limited data in this region. Although both the δ^18^O and δ^2^H cokriging isoscapes generally agree and predict isotope enrichment in the north east region (Fig 2), there could be isoscape digression undetectable in the individual isoscapes but amplified in the *d* isoscape (Fig 2e). Thus, there is need for more field observations for some of the locations to attain a better prediction model.

There is large variability in *d* across the Central Plateau in the cokriging isoscape (Fig 2e) and this can be attributed to the multiple sources of precipitation over this area as depicted in Fig 2a and 2c. The GFI *d* model, on the other hand, although not showing variability in the Central Plateau and eastern parts of Namibia depicting *d* > 10‰ for these areas (Fig 2f). This suggests either evaporation under low humidity or moisture recycling in the area [78, 79]. Therefore although different both *d* isoscapes demonstrate that *d* cannot be considered as a truly conservative tracer of environmental conditions at the vapour source as it could be significantly altered before the vapour arrives in Namibia [16, 23, 24] and because there are multiple sources of rain for Namibia.

### Rainfall isoscape validation

**Fig 4:**
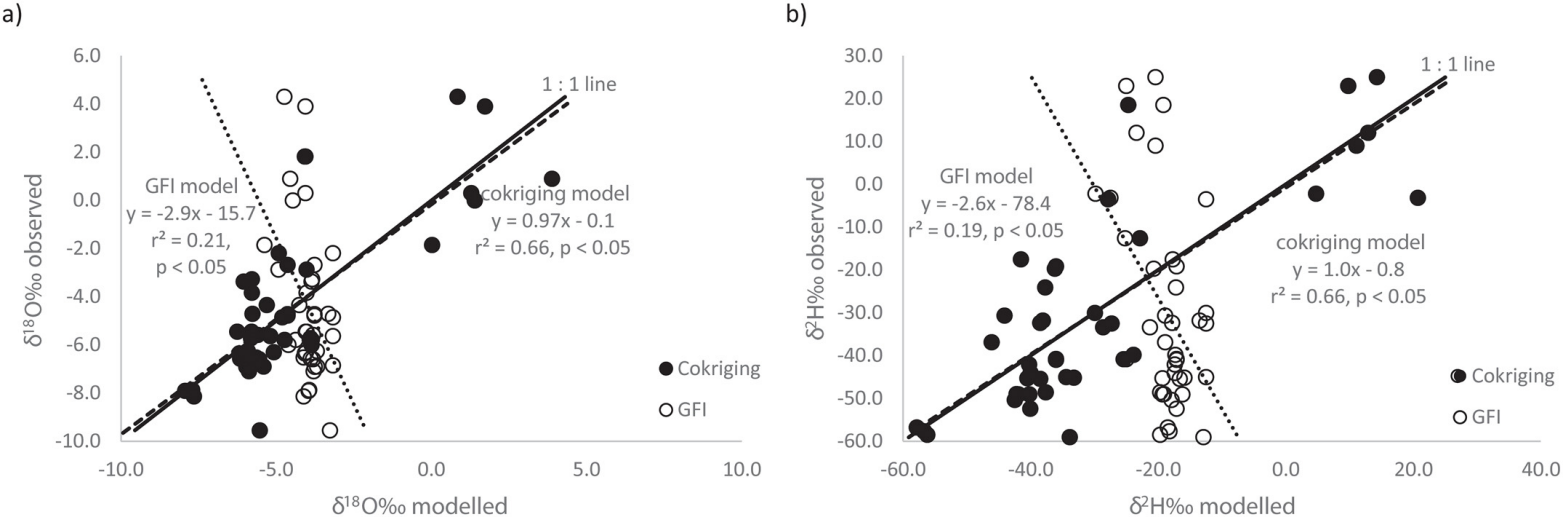
Rainfall cokriging and globally fitted isoscape (GFI) model validation using the observed data. (a) δ^18^O validation with 1:1 line as a reference; (b) δ^2^H validation with 1:1 line as a reference.

Because of the scarcity and quality (e.g., precipitation amounts were not reported) of data, apart from comparing the cokriging and GFI models to classic isotopic relationships as a means of evaluating the model performance, we also regressed unweighted observed isotopic values to the modelled data and compared the slope to the 1:1 line (Fig 4) [80]. The observed δ^18^O‰ values ranged between -9.6‰ and +4.3‰ (mean = -4.4‰), while the cokriging model values ranged between -7.9‰ and +3.9‰ (mean = -4.5‰) and those of the GFI model ranged between -5.4‰ and -3.2‰ (mean = -4.0‰) (Fig 4a). The observed δ^2^H‰ values ranged between -59‰ and +25‰ (mean = -30‰), while the cokriging model values ranged between -58‰ and +21‰ (mean = -30‰) and the GFI model values ranged between -30‰ and -13‰ (mean = -19‰) (Fig 4b). The modelled cokriging values of both δ^18^O‰ and δ^2^H‰ explain a larger proportion of the linear variance in the observed values (r^2^ = 0.67) in both cases compared to the GFI model (r^2^ = 0.17 and 0.19 for δ^18^O‰ and δ^2^H‰, respectively, Fig 4). The poor r^2^ between predicted and observed values of the GFI model (Fig 4) could be due to the fact that although the model was restricted to Namibia, it still reflected the global isotope distributions and not necessarily those specific to Namibia. We acknowledge the bias of the cokriging model towards the observed values because the model was not evaluated with an independent set of data because of data scarcity. However, the modelled cokriging values show expected large variations as the samples were collected from environmentally and climatically diverse locations (Figs 1 and 2) and closely follow the 1:1 line (Fig 4). The modelled values from the GFI model on the other hand do not correlate well with either of the observed δ^18^O‰ and δ^2^H‰ values (Fig 4) and do not show much variation despite the meteorological and physical differences in sampling locations (Fig 1). This suggests that the GFI model values are not a realistic estimate of the observed local values.

**Fig 5:**
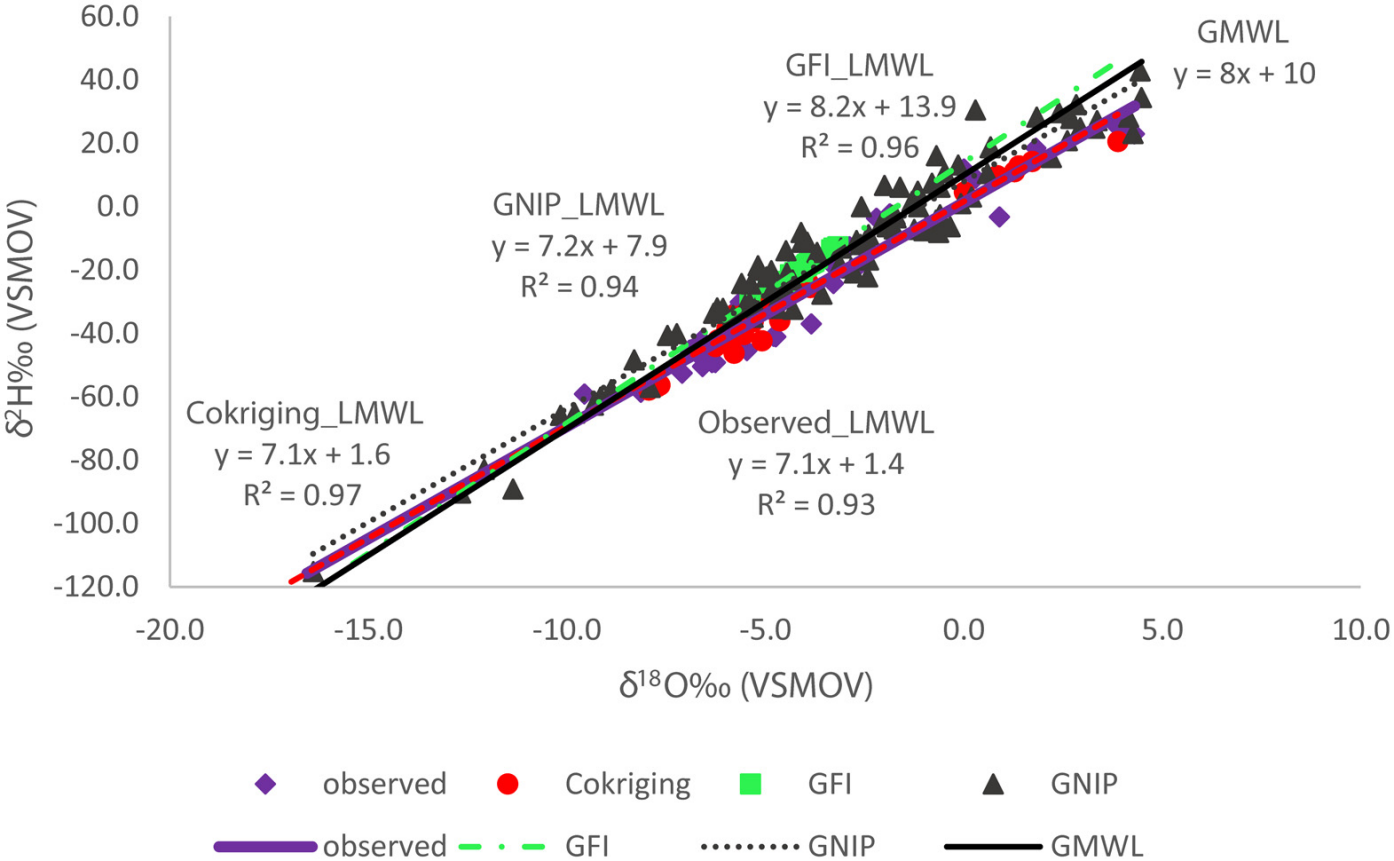
Modelled local meteoric water lines (LMWL) compared to observation-based LMWL with the global meteoric water line (GMWL) shown as a reference.

We used the GMWL (δ^2^H = 8 x δ^18^O +10) [81] to further validate our models. Because Namibia is classified as arid [82] we expect its local meteoric water line (LMWL) to plot below the GMWL (slope < 8). We thus constructed a LMWL based on data from the two Namibian GNIP stations (GNIP_LMWL) which had a slope of 7.2 consistent with our predictions (Fig 5). However, the GNIP_LMWL cannot be representative of the larger geographic area given that the data is from only two locations. Therefore, we calculated a second LMWL based on the observed data (Observed_LMWL) (slope 7.1), which was similar to the GNIP_LMWL with the only major difference being the intercept. However, the slopes point to some degree of aridity and this was similar to the modelled LMWL from the cokriging model (Fig 5). The GFI_LMWL was almost identical to the long term weighted mean GMWL defined as δ^2^H = 8.2 x δ^18^O + 10.35 [50, 81] but different from the Observed_LMWL. The GMWL [50] is a weighted global average constructed from the GNIP database. Therefore, the modelled Namibian GFI_LMWL reflects the average isotopic composition of global meteoric waters and does not account for local variations even when scaled down to a smaller geographic area. We cannot determine how much lower the slope would be because Namibia has different eco-regions ranging from hyper-arid desert to more subtropical (Fig 1) and the LMWL would intergrate these to provide an average which plots below the GMWL slope as that depicted by Observed_LMWL and the Cokriging_LMWL (Fig 5).

**Fig 6:**
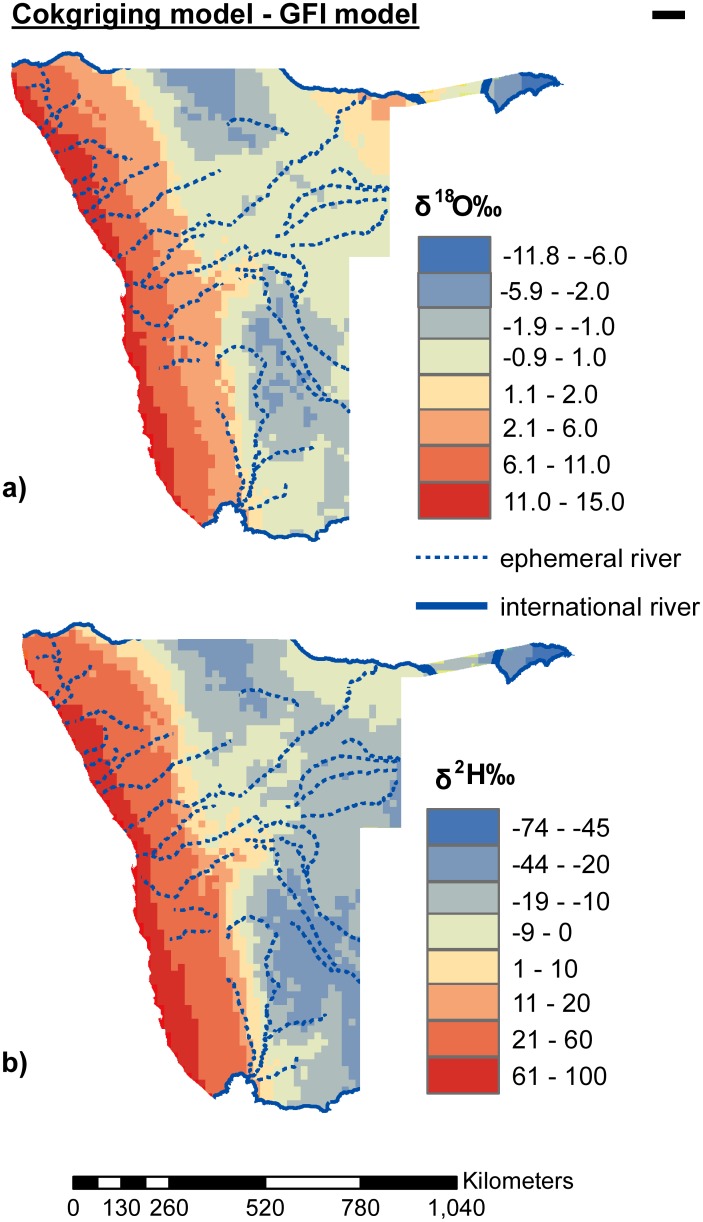
Calculated differences between the cokriging model and the globally fitted isoscape (GFI) model.

The differences between the cokriging and global models can be quantified to show areas of similarity and dissimilarity (Fig 6). The two models differ on their predictions of both δ^18^O and δ^2^H along the west coast varying by orders of magnitude 2–15‰ and 11–100‰ for δ^18^O and δ^2^H, respectively (Fig 6). These differences are largely because the two models exhibit different trends in this area with the cokriging model showing progressive rainfall isotopic depletion inland while the GFI model shows the opposite trend hence the maximum difference is observed along Namibia’s coast (Fig 6). In the north east (Zambesi region) there is a difference of -6.8 to -11.8‰ for δ^18^O between the models, which is also observed on the north central portions (Ohangwena and Oshikoto regions). The latter difference is because the cokriging model predicts depletion in this area due to rains originating from Angola along the TTTs while the GFI model predicts relatively enriched rains in the same area. Areas with ± 2‰ difference δ^18^O can be considered as areas of similar isotopic composition while areas of ±10‰ difference in δ^2^H similar in isotopic composition because the cokriging model uses unweighted averages while the global isoscape makes use of weighted averages (Fig 6).

## Conclusions

Rainfall isotopes across Namibia show coherent spatial distribution patterns that can be modelled and interpreted as isoscapes. Although global isoscape models are useful in depicting global trends of isotopic distributions they do not scale-down well and fail to capture or account for local variations. These global isoscapes often do not take into account local geographical features or meteorological parameters which could influence local rainfall patterns leading to low correlations between observed and modelled data. The relevance of global rainfall isoscapes to hydrologically interesting but under-represented regions is thus questionable. Although not ideal, the unweighted cokriging approach showed stronger r^2^ values with observed data (67% vs. < 24% for cokriging and GFI models, respectively) and this could be attributed to the inclusion of elevational data which generated local geographic features (Namib Escarpment) that have a profound effect on rainfall patterns across Namibia. The rainfall cokriging isoscapes also show that rainfall in Namibia is influenced by several synoptic weather systems originating from both the Atlantic and Ocean Oceans while the GFI isoscape suggests origins from the Indian Ocean alone. Therefore, although the absolute values may be subject to improvements; the trends of the local isoscape models appear more consistent with the synoptic systems affecting rainfall patterns in Namibia and the unweighted approach could be considered a viable alternative especially when dealing with historical data in data deficient regions.

## Supporting Information

S1 Table Rainfall database with isotopic compositions and associated physical and meteorological conditions of the sampling sites. (XLSX)
